# The role of ACER2 in intestinal sphingolipid metabolism and gastrointestinal cancers

**DOI:** 10.3389/fimmu.2024.1511283

**Published:** 2024-11-22

**Authors:** Binggang Liu, Junfeng Zhou, Biao Jiang, Bing Tang, Ting Liu, Pengcheng Lei

**Affiliations:** Department of Gastrointestinal Surgery, the Central Hospital of Yongzhou, Yongzhou, China

**Keywords:** ACER2, ceramidase, gastrointestinal tumors, sphingolipid metabolism, inflammation, immune modulation

## Abstract

Sphingolipids, particularly sphingosine-1-phosphate (S1P), are bioactive lipids involved in regulating cellular processes such as proliferation, apoptosis, inflammation, and tumor progression. Alkaline ceramidase 2 (ACER2) plays a critical role in sphingolipid metabolism by catalyzing the hydrolysis of ceramide to sphingosine, which is subsequently converted to S1P. Dysregulation of ACER2 has been implicated in various gastrointestinal cancers, including colorectal cancer, gastric cancer, and hepatocellular carcinoma. ACER2-mediated sphingolipid signaling, particularly through the SphK/S1P pathway, influences cancer development by modulating immune responses, inflammation, and the balance between cell survival and death. This review examines the physiological functions of ACER2, and its role in sphingolipid metabolism, and its contribution to the pathogenesis of gastrointestinal cancers. Understanding the mechanisms by which ACER2 regulates tumor progression and immune modulation may open new avenues for targeted therapies in gastrointestinal malignancies.

## 1 Introduction

Sphingolipids, including sphingomyelin (SM), ceramide (Cer), sphingosine (Sph), and sphingosine-1-phosphate (S1P), are a family of lipids with sphingoid bases. These molecules play an essential structural role in maintaining the fluidity and subdomain structure of lipid bilayers, especially lipid rafts (1). Enzymes such as ceramidase, ceramide synthase, sphingosine kinase (SphK), and S1P phosphatase metabolize sphingolipids, forming a network of metabolically related bioactive lipid mediators (2). Biochemical and molecular advances in sphingolipid metabolism have revealed that these sphingolipid metabolites also function as signaling molecules involved in regulating many cellular processes, including inflammation, proliferation, apoptosis, angiogenesis, and transformation (3–5). The role of sphingolipid metabolism in both normal and pathological states is gaining increasing recognition.

S1P, a sphingolipid metabolite, is a critical bioactive molecule in various aspects of cancer biology, including cell proliferation, migration, apoptosis, senescence, and inflammation (1). S1P was shown to affect inflammatory reactions and cancer development in various tissues (6–8), including the evolution and progression of gastrointestinal malignancies (9, 10). Sphingolipids, especially the S1P signaling axis, play different roles in regulating innate and adaptive immunity, immune surveillance, immune cell trafficking and differentiation, cytokine release, and endothelial barrier integrity through S1P binding to S1P receptors (S1PR) ubiquitously expressed in gastrointestinal tissues, and are involved in regulating inflammation-related responses in normal and malignant gastrointestinal cells and tissues (11). The link between inflammation and gastrointestinal (GI) malignancies is well established (12–14). However, the role of sphingolipid signaling in mediating proinflammatory responses and GI cancer development has not been adequately addressed. Given the unique exposure of the GI tract to dietary sphingolipids and their associated enzymes, the relationship between sphingolipids and GI inflammation and tumors is a novel and complex issue. Alkaline ceramidase ACER2 has been identified as a key regulator of circulating S1P levels (2, 15), but its role in the development of gastrointestinal tumors is not fully understood. This review will focus on the physiological role of ACER2 and its role in sphingolipid metabolism and GI cancer, this will provide new strategies to prevent GI cancer progression.

## 2 ACER2 involves in sphingolipid metabolism, inflammation and cancer

### 2.1 Classification, distribution, and function of ceramidases

Ceramidases are key to the degradation of ceramide into sphingosine and free fatty acids in the sphingolipid pathway (16), contributing to the homeostasis of Cer and S1P. Five ceramidases have been identified in humans, classified as acidic ceramidase (AC), neutral ceramidase (nCDase), and alkaline ceramidases 1-3 (ACER1, ACER2, and ACER3) (17). This classification reflects the optimal pH for each enzyme’s catalytic activity (18, 19). AC is located in the lysosomal compartment, and congenital deficiency of AC results in Farber disease. AC is also involved in regulating cell viability and response to stressors, particularly chemotherapeutic drugs (20). Neutral ceramidase is primarily localized to the plasma membrane (but also to the Golgi apparatus and mitochondria), is mainly expressed in the small intestine and colon, and is associated with digestive processes and colonic carcinogenesis (21). ACER1-3 belong to a closely related family initially identified in yeast (22, 23). ACER1 is present in the endoplasmic reticulum and is highly expressed in the skin, where it plays a critical role in keratinocyte differentiation (24). ACER2 is localized to the Golgi complex, highly expressed in the placenta, and involved in programmed cell death in response to DNA damage (25). ACER3 is also localized in the endoplasmic reticulum and Golgi complex, broadly expressed, and is associated with Purkinje cell degeneration, contributing to motor coordination (26).

### 2.2 ACER2 in sphingolipid metabolism

ACER2 is a 31 kDa membrane protein composed of 275 amino acids, with seven presumed transmembrane domains allowing its association with the Golgi apparatus (27). The Michaelis-Menten constant (Km) values of ACER2 for ceramide are approximately 94.8–98.5 µM, depending on the substrate derivative used (28). ACER2 is expressed in various tissues, including the placenta, pancreas, and heart (27). The expression of ACER2 is regulated by tumor suppressor p53 and hypoxia-inducible factor 2α (29–32). Elevated ACER2 mRNA expression has been observed in human cancer tissues, including liver and colon cancers, compared to healthy samples (27). Under stress conditions, such as exposure to glucocorticoids or reactive oxygen species (ROS), sphingosine produced by ACER2 induces programmed cell death by increasing ROS production in response to DNA damage (29).

ACER2 catalyzes the hydrolysis of ceramide into sphingosine, which is phosphorylated by SphK (SphK1 and SphK2) to form S1P (33). Upregulation of ACER2 leads to increased levels of sphingosine and S1P in cells, while ceramide levels are reduced. Ceramide is known as an antiproliferative, pro-apoptotic, and pro-senescent bioactive lipid (3, 34, 35). Sphingosine also mediates cell cycle arrest, differentiation, and programmed cell death (PCD) (24, 27, 29, 36–38). In contrast, S1P promotes cell proliferation and survival and inhibits senescence (39). S1P is most abundant in the intestine, the activation of the mitogen-activated protein kinases (MAPKs) is among the best-characterized S1P effects. Because the MAPKs regulate proliferation, S1P stimulate intestinal epithelial cell proliferation by MAPK activation (40). S1P induces cyclooxygenase-2 (COX-2) expression via PI3K/Akt and p42/p44 MAPK pathways in rat vascular smooth muscle cells (41). Besides, S1P mediates COX-2 expression and prostaglandin E2 (PGE2)/IL-6 secretion via c-Src-dependent AP-1 activation, thus promotes airway inflammation (42). The binding of critical regulator YTH N6-methyladenosine RNA binding protein 2 (YTHDF2) to m6A sites on ACER2 mRNA promoted its stability and expression. Enhanced ACER2 expression hydrolyzed ceramides, disrupting the balance between Cer and S1P, activating the ERK and PI3K/AKT pathways, and leading to diffuse large B-cell lymphoma (DLBCL) tumorigenesis (43). In addition, S1P is enriched in the blood and lymph (44), and the S1P gradient in blood vessels is used to regulate immune cell trafficking, including lymphocytes, hematopoietic progenitor cells, and dendritic cells (45).

### 2.3 ACER2-S1P signaling axis: effects on immune response and cancer progression

S1P acts as a ligand for a group of five G protein-coupled receptors known as S1PR1–5. The S1P1–3 receptors are highly expressed in many tissues, particularly within the cardiovascular and immune systems, while S1PR4 is more selectively expressed in the lymphatic system and airway smooth muscle cells (46, 47). S1PR5, on the other hand, is predominantly found in the white matter of the central nervous system (48, 49), though the expression of S1PR4 and S1PR5 is comparatively lower than that of S1PR1–3. These S1P receptors play a critical role in regulating immune cell trafficking and cancer progression by influencing cell migration, proliferation, and survival (45).

S1P signaling has been shown to regulate the trafficking of various immune cells, including dendritic cells (50), natural killer (NK) cells (51, 52), T cells (53, 54), and hematopoietic stem cells (55). Additionally, S1P exhibits strong pro-inflammatory effects, acting as a chemoattractant for neutrophils and macrophages (56). Within macrophages, SphK1 is essential for complement component C5a-induced intracellular calcium mobilization, degranulation, and the production of cytokines such as TNF-α, IL-6, and IL-8, as well as for chemotaxis (57). SphK1 also mediates TNF-α-induced PGE2 synthesis (58). Research indicates that TNF-triggered transcription of proinflammatory cytokines, chemokines, and adhesion molecules, including IL-6, RANTES, MCP-1, and VCAM-1, requires SphK1 activation (59). In endothelial cells, S1P has been found to inhibit TNF-α-driven monocyte adhesion both *in vitro* and *in vivo* (60, 61). Activation of SphK1 by TNF-α leads to S1P production, which subsequently activates S1P1 and S1P3 through autocrine signaling, enhancing nitric oxide production by eNOS (62). Nitric oxide, in turn, reduces the expression of adhesion molecules and the adhesion of leukocytes (63–65).

External S1P, when added to NK cells, binds to S1P receptors, inducing cell chemotaxis (66). Mice lacking S1P exhibit reduced NK cell migration and impaired efflux (67). The mobilization of NK cells relies on S1PR5 signaling (68), and further studies indicate that the binding of S1PR5 to CXCR4 is essential for NK cell mobilization and the rapid production of interferon following activation (69). The exit of CD4^+^ T cells from lymph nodes is dependent on the S1PR1/CD69 axis, and S1P upregulates adhesion molecules crucial for T cell recruitment (54). Both S1PR1 and S1PR4 exhibit chemotactic effects on T cells (70). While S1P facilitates T cell movement, it also inhibits PMA-induced T cell proliferation *in vitro* (71).

S1P is known to facilitate cancer progression through several mechanisms (72–74). Elevated S1P concentrations have been observed in various cancer types, such as breast, gastric, and pancreatic cancers, where levels within tumor tissues consistently surpass those in adjacent non-tumor tissues (75–78). Recent studies have demonstrated a key role for sphingolipid metabolic pathways in liver regeneration, hepatocellular carcinoma (HCC) progression, and treatment (79, 80). Previous research has identified S1P as the pivotal element connecting chronic inflammation to cancer progression, particularly in colitis-associated cancers (81). It plays a central role in the NF-κB-regulated production of IL-6 and the sustained activation of STAT3, which, in turn, elevates S1PR1 expression (82). This S1P/S1PR1/STAT3 signaling axis establishes a feed-forward loop that exacerbates chronic inflammation, thus contributing to disease progression. Furthermore, the production of S1P by cancer cells at high levels enhances the tumor microenvironment, reinforcing the connection between cancer and inflammation (83). Sphingolipids play a crucial role in regulating inflammation and extracellular matrix dynamics in the tumor microenvironment (84). S1P can activate various signaling pathways in immune cells (85), endothelial cells (86), and fibroblasts (87, 88), affecting the secretion of cytokines, chemokines, and growth factors that regulate inflammation (89, 90). In addition, S1P interacts with its receptor S1PR1-5, coordinating a multifaceted regulatory network and affecting the dynamic balance of cytotoxic T cells, Tregs, Th17 cells, and NK cells (89), thus affecting tumor progression and the efficacy of immunotherapy by regulating the tumor immune microenvironment.

ACER2 has been identified as a transcriptional target of p53, ACER2 activation by p53 increases reactive oxygen species (ROS) production, thereby mediating the DNA damage response (29, 91). In various cancer cell lines, ACER2 overexpression influences key processes such as cellular proliferation, DNA damage response, programmed cell death, and autophagy (29). Notably, ectopic expression of ACER2 exhibits a dual effect on tumor cell dynamics, promoting both proliferation and apoptosis. Xu et al. demonstrated that ACER2 enhances the synthesis of S1P, which is associated with cell proliferation and survival; however, excessive ACER2 levels may lead to cell cycle arrest due to sphingosine accumulation (27). Additionally, ACER2 levels show a negative correlation with several well-characterized immune checkpoint inhibitors, which may hinder the re-activation of tumor-specific cytotoxic T lymphocytes responsible for targeting and eliminating cancer cells (92, 93).

## 3 ACER2 in GI cancers

Lipids *de novo* transformed and biosynthesized by the intestinal microbiome have important structural and signaling functions, which can affect host cells through metabolic and immune pathways (94). The host can directly sense microbial-derived lipids, thereby regulating innate and adaptive immune pathways and regulating metabolic pathways (95). Mammalian sphingolipid signaling is essential for many inflammatory and cell survival pathways and plays an important role in many metabolic and inflammatory diseases (96). Changes in membrane phospholipid chemistry can lead to increased intestinal permeability, allowing bacteria to spread in the host, resulting in many pathological consequences (97). In addition, microbial-derived SP deficiency is associated with inflammatory bowel disease (IBD) (94). Sphingolipids are an integral part of tumor lipid metabolism, which significantly influences cancer progression, metastasis, and drug resistance (98, 99). In addition, the metabolic pathways of sphingolipids, which include the synthesis and degradation of these complex molecules, are tightly linked to several key signaling pathways that drive the carcinogenic process (72, 100). For example, the balance between intracellular ceramide, ceramide-1-phosphate (C1P), sphingosine, S1P, and glycosphingolipids can determine the balance between cell death and survival, thus significantly affecting tumor progression and treatment outcomes (72, 100).

### 3.1 ACER2 and colorectal cancer

The development of colorectal cancer (CRC) is accompanied with complicated alterations of the metabolism of sphingolipids in cancer tissue (101). Mazzei et al. reported that dietary SM modulates the inflammatory response in the early stages of azoxymethane (AOM)/dextran sulfate sodium (DSS)-induced colon cancer by activating peroxisome proliferator-activated receptor γ (PPAR-γ) (102). Sphingolipid metabolism forms a network of metabolically related bioactive lipid mediators. The critical role of S1P signaling in gastrointestinal tumors has been highlighted in previous research (103). Sph can be phosphorylated by SPHK 1 and SPHK 2 in enterocytes to form S1P, which can inhibit apoptosis, promote proliferation and angiogenesis, and induce inflammatory signaling by activating the nuclear factor κB (NF-κB) and STAT3 pathways (70, 84). The epithelial STAT3-S1P axis is thought to influence tumor progression by modulating the recruitment of distinct immune cell populations. Proinflammatory cytokines, such as IL-6, secreted by infiltrating inflammatory cells, initiate STAT3 activation within epithelial cells. Beyond supporting pathways that control cell survival and proliferation, epithelial STAT3 also activates the SphK-S1P-S1PR cascade, which further amplifies epithelial STAT3 activation by (1) facilitating the recruitment of inflammatory cells, including CD8^+^ T cells and regulatory T cells (Tregs), and (2) reinforcing the positive feedback loop mediated by S1PR-STAT3 signaling in epithelial cells. This self-sustaining loop drives persistent STAT3 activation in epithelial cells, ultimately contributing to their malignant transformation (104).

Besides, previous studies have shown that the SphK1/S1P pathway mediates the arachidonic acid (AA) cascade (105), especially inducible COX-2 and its product, the inflammatory mediator PGE2, which is associated with colon cancer (1, 106, 107). SphK1 is involved in acute colitis (108), intestinal polyp formation (103), and colon carcinogenesis (109). SphK2 may also be involved in DSS-induced acute colitis and AOM/DSS-induced colitis-driven colon carcinogenesis (110, 111). Besides, The increase in S1P/Cer ratio is related to the increased tumor cells survival, growth, and progression in colon cancer (112). ACER2 plays a key role in SphK/S1P signaling-mediated colon inflammation and cancer development. Besides, studies have shown that ACER2 activity can increase the levels of Sph and S1P while reducing the levels of Cer, and this activity has been shown to be involved in the p53-mediated DNA damage response and the regulation of cell cycle arrest and cell senescence in colon cancer cells (31).

### 3.3 ACER2 and GC

ACER2 has also been implicated in gastric cancer. miRNAs can interact with 3′-UTR, 5′-UTR, coding sequences and gene promoters to regulate gene expression; the most common is to target 3′-UTR to inhibit gene expression (113). miR-196a-5p is associated with the progression from chronic atrophic gastritis to gastric cancer (GC) and promotes the malignant behavior of GC cells by directly targeting the 3’UTR of ACER2 mRNA, reducing its expression (114). The suppression of ACER2 expression leads to an imbalance between sphingosine and S1P levels, promoting cell proliferation (114). When cells express low levels of ACER2, the proliferative effects of S1P can offset the antiproliferative effects of low levels of Sph, thereby promoting cell proliferation, whereas in cells expressing high levels of ACER2, the apoptotic effects of high levels of Sph can override the anti-apoptotic effects of S1P, leading to PCD (31). Additionally, sphingolipids, including ceramide and S1P, play a role in viral infections such as Epstein-Barr virus (EBV), which is associated with GC. Recent studies suggest that ceramide promotes the lytic reactivation of EBV in GC, further highlighting the complex interplay between sphingolipid metabolism and viral oncogenesis in GC (115).

### 3.4 ACER2 and HCC

ACER2 has been implicated in hepatocellular carcinoma (HCC) as well. In mice, MmACER2 was identified as CRG-L1, a gene linked to liver cancer, and its expression was elevated in hepatocellular carcinoma within a mouse model induced by diethylnitrosamine, a known carcinogen (2). This upregulation was observed across three stages—quiescent, regenerating, and neoplastic liver—when compared to normal liver tissue (116). Similarly, HsACER2 showed increased expression in HCC tumors and cell lines relative to non-tumor tissue in both patients and the non-tumorigenic human hepatocyte line QSG-7701. The upregulation of HsACER2 contributed to enhanced proliferation of HCC tumor cells *in vitro*, stimulated HCC growth in xenograft models, and promoted both migration and invasion of HCC (117).

**Figure 1:**
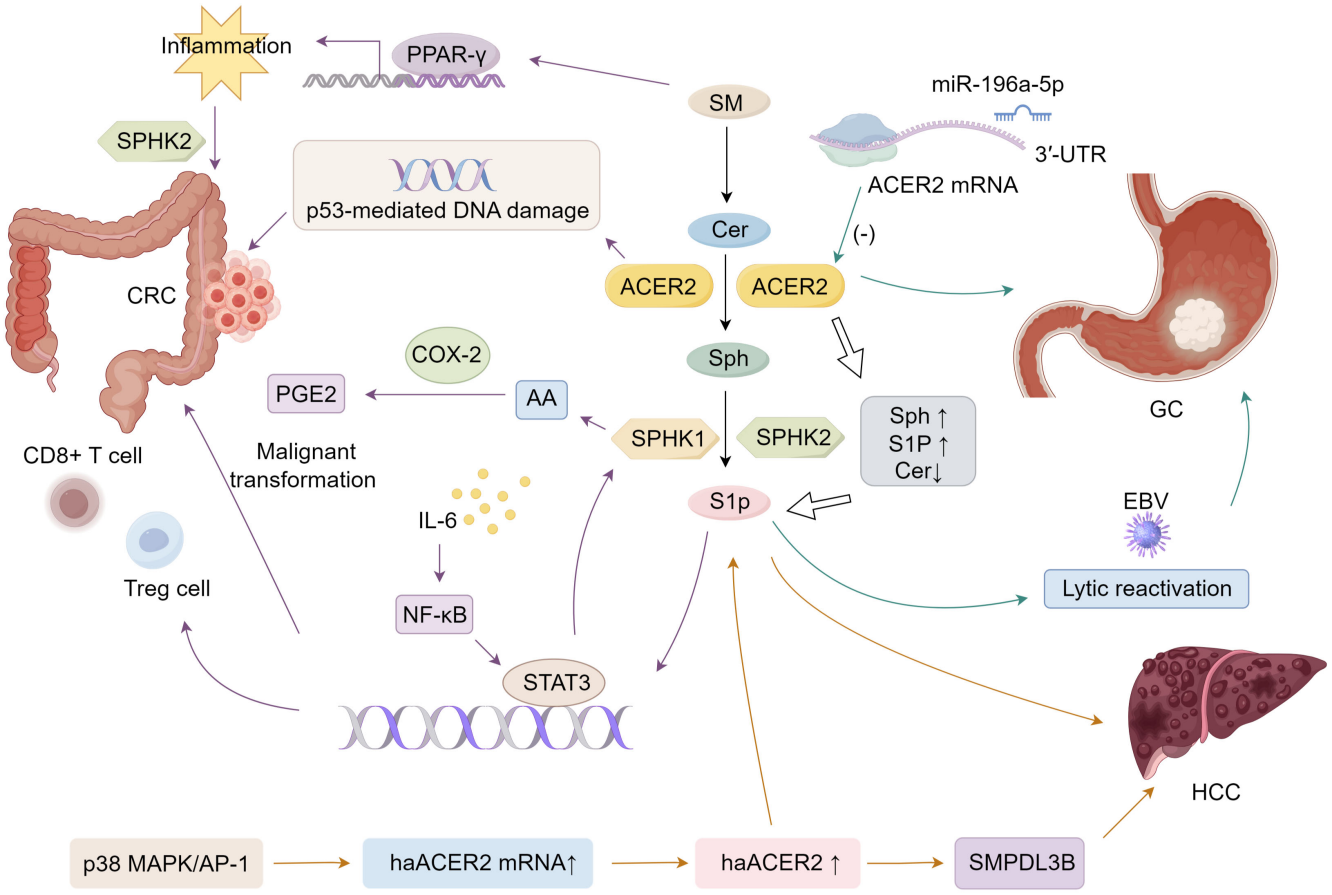
Mechanisms of ACER2 that facilitates GI cancers progression.

The activity of haCER2 was found to be upregulated in HepG2 human hepatoma cells under conditions of serum deprivation. This increase resulted from elevated haCER2 mRNA levels, driven by mRNA transcription rather than mRNA stability (118). The p38 MAPK/AP-1 signaling pathway was implicated in the upregulation of haCER2 mRNA during serum deprivation, providing a possible mechanism for haCER2 elevation in human HCC (118). Notably, elevated haCER2 expression triggered sphingosine-induced growth arrest, while its lower expression favored S1P-driven cell proliferation (27). Thus, the regulation of haCER2 likely plays a crucial role in determining cell fate by modulating the balance between ceramide/sphingosine and S1P levels. Moreover, ACER2 facilitates HCC cell proliferation, invasion, and migration through mechanisms involving sphingomyelin phosphodiesterase acid-like 3B (SMPDL3B) (117) (**Figure 1**).

## 4 Conclusion

ACER2 plays a pivotal role in regulating sphingolipid metabolism, influencing key processes such as cell proliferation, apoptosis, and immune modulation. Its dysregulation has been implicated in a range of gastrointestinal cancers, including CRC, GC, and liver cancer. By modulating the balance between pro-apoptotic ceramide, pro-survival sphingosine, and S1P, ACER2 contributes to both the promotion and suppression of cancer progression, depending on the context of its expression and regulation (117, 119).

The involvement of ACER2 in sphingolipid metabolism positions it as a promising therapeutic target in GI cancers (120), particularly in cancers where its expression is dysregulated. Future research focusing on the precise mechanisms by which ACER2 contributes to tumor progression and immune modulation will provide valuable insights into novel treatment strategies for GI cancers.
